# Notch-RBP-J-Independent Marginal Zone B Cell Development in IgH Transgenic Mice with V_H_ Derived from a Natural Polyreactive Antibody

**DOI:** 10.1371/journal.pone.0038894

**Published:** 2012-06-13

**Authors:** Zhuo Zhang, Lanhua Zhou, Xinwei Yang, Yaochun Wang, Ping Zhang, Lihong Hou, Xinbin Hu, Ying Xing, Yufeng Liu, Wei Li, Hua Han

**Affiliations:** 1 Department of Dermatology, Xijing Hospital, Fourth Military Medical University, Xi'an, China; 2 State Key Laboratory of Cancer Biology, Department of Medical Genetics and Developmental Biology, Fourth Military Medical University, Xi'an, China; 3 Department of Neurology, Tangdu Hospital, Fourth Military Medical University, Xi'an, China; 4 Department of Dermatology, Affiliated Hospital of Institute of Aviation Medicine, Air Force, Beijing, China; Institut Pasteur, France

## Abstract

Both the B cell antigen receptor (BCR) signaling and Notch signaling pathway play important roles in marginal zone (MZ) B cell development; however, if and how these two signaling pathways engage in crosstalk with each other remain unclear. In the present study, IgH transgenic mice (TgV_H_3B4) were crossed with mice with Notch downstream transcription factor RBP-J floxed alleles (RBP-J^f/f^) and Mx-Cre transgene. Subsequently, MZ B cell development was analyzed in 3B4/Cre/RBP-J^f/f^ mice that expressed the transgenic 3B4 IgH and exhibited a deficiency in Notch signaling in B cells upon poly (I:C) injection. We observed that MZ B cell numbers were severely reduced, but still detectable in 3B4/Cre/RBP-J^f/f^ mice, in contrast to increased numbers of MZ B cells in TgV_H_3B4 mice and almost no MZ B cells in Cre/RBP-J^f/f^ mice. The majority of the MZ B cells in the 3B4/Cre/RBP-J^f/f^ mice had the same antigen specificity with that of 3B4 antibody, indicating that a particular BCR specificity might direct MZ B cell development in the absence of Notch signaling. The number of MZ B precursor (MZP) cells was reduced sharply in 3B4/Cre/RBP-J^f/f^ mice, and the number of transitional stage 1 and transitional stage 2 cells did not change that much, indicating that the interaction between BCR and Notch signaling likely occurred during the T2-MZP stage. Based on the transgenic mouse model, our data indicate that MZ B cells with certain BCR specificity can develop in a Notch-RBP-J independent manner.

## Introduction

Marginal zone (MZ) B cells are a separate B cell lineage distinct from mature follicular (FO) B cells and B-1 cells, and are critical determinants of the host defense against blood-borne bacterial pathogens [1], [2]. MZ B cells reside in the outer white pulp of the spleen between the marginal sinus and the red pulp; they are identified as being CD21^hi^CD23^low^ CD9^+^CD1d^hi^IgD^low^IgM^hi^ [3], [4]. MZ B cells are generated as naïve B cells that intrinsically possess several properties that resemble those of memory B cells, including a pre-activated phenotype and the ability to self-renew and survive for the duration of the life span of the host [1], [2], [5].

The mechanisms underlying MZ B cell development remains unclear. B cells undergo a series of differentiation checkpoints before they become mature functional antibody-secreting cells [6], [7]. After B lineage commitment, progenitor B cells (pro-B) begin to rearrange their B cell antigen receptor (BCR) genes at the V_H_ loci and differentiate into precursor B (pre-B) cells [8]. Next, pre-B cells undergo a second round of BCR rearrangement at the V_L_ loci to generate immature B cells that possess a functional BCR [9]. These immature B cells next undergo negative selection, during which B cell clones that respond to self-antigens are cleared through apoptosis, anergy, or receptor editing [10]–[12]. Immature B cells that are weakly or not self-reactive mature further. Newly generated B cells that have yet to acquire the ability to recirculate are known as transitional stage 1 (T1) B cells. Upon entering the follicles, these cells acquire surface IgD and CD23 expression while maintaining the expression of markers of immaturity. These cells are considered transitional stage 2 (T2) B cells [13], [14]. T2 B cells then differentiate directly into FO B cells or pass through the T2-MZ B cell progenitor (MZP) stage into the MZ B cell compartment [2].

Several signaling pathways triggered by cell surface receptors are required for MZ B cell development, including BCR signaling [15]–[17], Notch signaling [18], [19], canonical and non-canonical NF-κB signaling [20], [21], and BAFFR signaling [22]–[24]. BCR signaling is the fundamental driving force for MZ B cell development. It has been well documented that weak BCR signaling leads B cells to develop into MZ B cells. For example, mutations of molecules that attenuate BCR signaling, such as Aiolos or CD22, result in an increased number of FO B cells, while MZ B cells are absent [25], [26]. Conversely, deficiencies in molecules that reduce BCR signaling, such as CD21/CR2 deficiency, increase the number of MZ B cells [25]. Additionally, increased Btk signaling promotes FO B cell development, while weakening of this signaling pathway promotes MZ B cell development [2]. Certain BCR specificities also drive MZ B cell development. In V_H_81X transgenic mice, B cells differentiate into MZ B cells, while in anti-hen egg lysozyme and anti-class I MHC transgenic mice, B cells differentiate into FO B cells [27], [28]. However, it has been suggested that once a cell has been selected to become a FO B cell or a MZP B cell based on BCR specificity and signaling strength, further development into a mature FO or a MZ B cell, respectively, does not occur by default [29]. Signaling from other pathways, such as Notch2-mediated signaling, is required for MZ B cell generation.

The mammalian Notch family consists of four highly conserved transmembrane receptors that regulate cell fate determination in various tissues [30]. Ligand binding to Notch receptors leads to proteolytic processing within the transmembrane domains and the subsequent release of the intracellular domain [31], [32]. The Notch intracellular domain then translocates into the nucleus, where it forms a ternary complex with RBP-J/CBF1 (the vertebrate homolog of Suppressor of Hairless) and Mastermind, a transcriptional coactivator, to mediate gene transcription activation [33]. The role of Notch signaling in the development of MZ B cells has been firmly established. When RBP-J is conditionally deleted in B cells, MZ B cells are absent and FO B cell numbers are enhanced [18]. Also, conditional knockout studies revealed that Delta-like1 (Dll1) [19], Notch2 [34] and Mastermind-like (MAML) proteins are all required for MZ B cell development.

Although both BCR and Notch signaling pathways are required for MZ B cell development, if and how these two signaling pathways interact with each another remains unknown. Previously, we generated IgH transgenic mice TgV_H_3B4 with the V_H_ derived from the natural autoantibody producing hybridoma 3B4. These mice exhibited an increased proportion of MZ B cells [35], [36]. In the present study, TgV_H_3B4 mice, transgenic mice containing a floxed allele of RBP-J (RBP-J^f/f^) and Mx-Cre mice were crossed to obtain mice that have the transgenic IgH, but deficient in Notch signaling in B cells following the conditional deletion of RBP-J. The results demonstrated that there were increased numbers of MZ B cells in TgV_H_3B4 mice and almost no MZ B cells in Cre/RBP-J^f/f^ mice. In 3B4/Cre/RBP-J^f/f^ mice, although MZ B cell numbers were substantially reduced, MZ B cells were still detectable. The majority of the MZ B cells in the 3B4/Cre/RBP-J^f/f^ mice had the same antigen specificity with that of 3B4 antibody, indicating that the abrogation of Notch-RBP-J signaling in B cells can attenuate BCR-directed MZ B cell development, likely by reducing BCR diversity.

## Results

### Abrogation of Notch signaling attenuated MZ B cell development in IgH transgenic mice

To induce the deletional inactivation of RBP-J through Cre expression in the IgH transgenic mice TgV_H_3B4, mice containing the floxed RBP-J allele were bred with TgV_H_3B4 mice and Mx-Cre transgenic mice in which Cre recombinase expression is transactivated by the Mx promoter after the IFN-α inducer ploy (I:C) injection [37]. Mice with the Cre/RBP-J^f/+^, 3B4/Cre/RBP-J^f/+^, Cre/RBP-J^f/f^ and 3B4/Cre/RBP-J^f/f^ genotypes were analyzed after the injection of poly (I:C). Consistent with a previous report [37], deletion of the RBP-J allele was nearly 100% in the bone marrow (BM) and approximately 80% in the spleen as confirmed using Southern blot analysis (data not shown).

**Figure 1:**
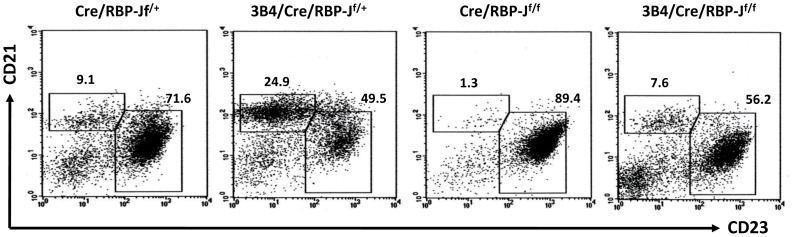
Analysis of MZ and FO B cells following Mx-Cre-induced RBP-J deletion. Mice with genotypes of Cre/RBP-J^f/+^, 3B4/Cre/RBP-J^f/+^, Cre/RBP-J^f/f^ and 3B4/Cre/RBP-J^f/f^ were compared. Deletion of the floxed RBP-J exons was induced through the injection of poly(I:C); mice were then sacrificed and subjected to MZ and FO B cell analysis by FCM. The cells were first gated by anti-CD19 and then CD21 and CD23 expression was utilized to discriminate between FO (CD19^+^CD21^int^CD23^hi^) and MZ B (CD19^+^CD21^hi^CD23^lo^) cells. The numbers denote the percentages of cells in the indicated squares. Data are representative of five independent experiments.

Next, we analyzed B cell development in the spleen following RBP-J deletion. Anti-CD19, anti-CD21 and anti-CD23 antibodies were used to discriminate between FO B (CD19^+^CD21^int^CD23^hi^) and MZ B (CD19^+^CD21^hi^CD23^lo^) cells. Consistent with previous results [35], [36], we observed an increased proportion of MZ B cells in 3B4/Cre/RBP-J^f/+^ mice, which was predominately due to decreased FO B cell production (Figure 1). Also in agreement with earlier findings, the MZ B cell proportion was significantly reduced in the Cre/RBP-J^f/f^ mice because of the abrogated Notch signaling (Figure 1) [18]. Interestingly, the population of MZ B cells in the 3B4/Cre/RBP-J^f/f^ mice was greatly reduced compared with the 3B4/Cre/RBP-J^f/+^ mice; however, the proportion of MZ B cells in 3B4/Cre/RBP-J^f/f^ mice was still much higher compared with the Cre/RBP-J^f/f^ mice (Figure 1). There was no difference in phenotype between wild-type and the Cre/RBP-J^f/+^ mice. These data indicate that abrogation of Notch signaling attenuated but not abrogated the MZ B cell differentiation in 3B4 IgH transgenic mice.

### A small population of MZ B cells exist in the absence of RBP-J in IgH transgenic mice

**Figure 2:**
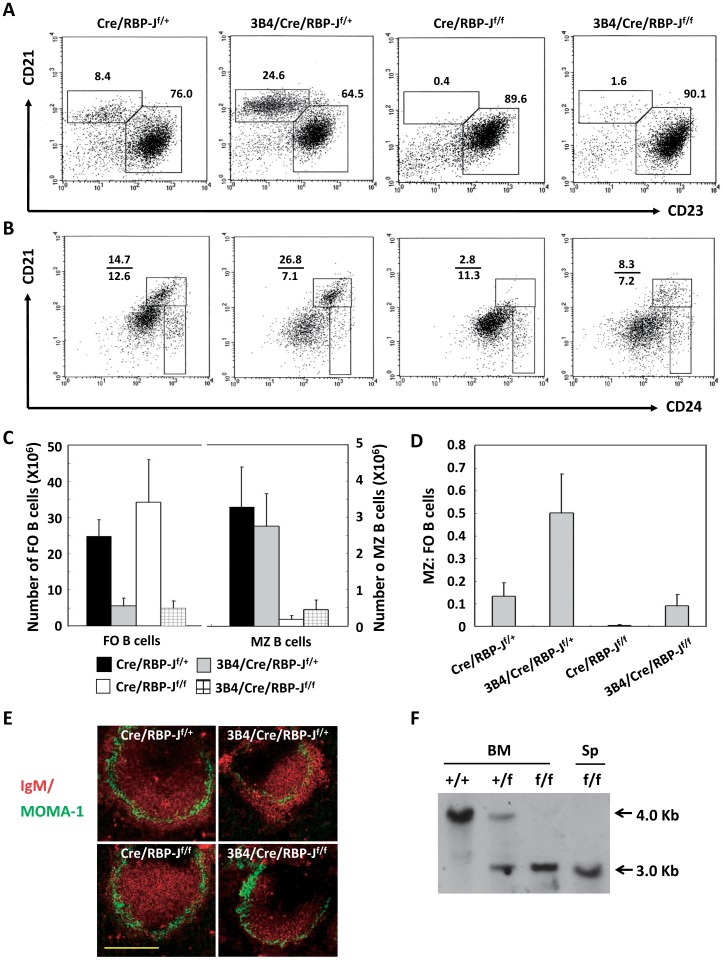
Analysis of MZ and FO B cells following BM transplantation. (A) and (B) Two months following BM transplantation, the four groups of mice were sacrificed and spleen cells were stained using anti-CD19, anti-CD21 and anti-CD23 (A), or anti-CD19, anti-CD21 and anti-CD24 (B) and then analyzed using FCM. The cells were initially gated using anti-CD19. CD24^hi^CD21^hi^ cells represent MZ/T2 B cells, and CD21^med^CD24^hi^ cells represent T1 cells. The numbers denote the percentages of cells in the indicated squares. Data are representative of five independent experiments. (C) Absolute numbers of MZ and FO B cells. Numbers were calculated by multiplying the fraction of each FCM B cell subset analyzed in (A) by the total number of CD19^+^ cells. Data are mean±S.D. obtained from five independent experiments. (D) The ratio of MZ to FO cell number was calculated. (E) Immunofluorescent staining of splenic cryosections with Cy3-anti-IgM and FITC-anti–MOMA-1. MOMA-1^+^ metalophilic macrophages define the border between the MZ B and FO B cells. Data are representative of five independent experiments. Scale bar, 100 µm. (F) Southern blot analysis of the DNA from the BM and spleen of the recipient mice. 4 Kb fragment represents wild-type, and 3 Kb fragment represent deletion.

BM transplantation was utilized to further analyze the MZ B cell development. This methodology was selected because poly(I:C) injection induced the non-specific activation of many cell types; further, the deletion efficiency of the RBP-J allele only reached 80% in the spleen following poly(I:C) injection. Two months after BM transplantation, southern blot was applied to analyze the DNA from the BM and spleen of the recipient mice. The result showed that the RBP-J allele was deleted completely in both BM and spleen after BM transplantation (Figure 2F). Then the recipient mice were sacrificed and spleen cells were prepared for flow cytometric (FCM) analysis. As described previously, there were elevated frequency of MZ B cells in 3B4/Cre/RBP-J^f/+^ mice; further, we observed almost no MZ B cell subset in Cre/RBP-J^f/f^ mice (Figure 2A). Although the proportion of MZ B cells was significantly decreased in 3B4/Cre/RBP-J^f/f^ mice, it was higher compared with Cre/RBP-J^f/f^ mice (Figure 2A). When analyzed using anti-CD21 and anti-CD24 antibodies, similar results were obtained. While the proportion of CD21^hi^CD24^hi^ MZ/T2 cells was elevated in 3B4/Cre/RBP-J^f/+^ mice and significantly reduced in Cre/RBP-J^f/f^ mice, the frequency of MZ/T2 cells in 3B4/Cre/RBP-J^f/f^ mice was lower than 3B4/Cre/RBP-J^f/+^ mice and higher than Cre/RBP-J^f/f^ mice (Figure 2B). In agreement with flow cytometric data, histological examination of the spleen showed the loss of MZB cells in Cre/RBP-J^f/+^ mice and 3B4/Cre/RBP-J^f/f^ mice by staining of the MOMA-1^+^ metalophilic macrophages that define the border between the MZ B and FO B cells (Figure 2E). This defect was observed in all follicles of the mice.

The total numbers of FO and MZ B cells were estimated from FCM profiles and the total number of CD19^+^ cells (Figure 2C). In 3B4/Cre/RBP-J^f/+^ mice, we observed a four- to five-fold decrease in the number of FO B cells, while the MZ B cell numbers remained unchanged. In Cre/RBP-J^f/f^ mice, we observed a slight increase in FO B cell numbers and MZ B cells were almost absent. However, in 3B4/Cre/RBP-J^f/f^ mice, both FO and MZ B cell numbers were reduced; importantly, the number of MZ B cells in 3B4/Cre/RBP-J^f/f^ mice was still elevated compared with Cre/RBP-J^f/f^ mice. The ratio of MZ B cells to FO B cells was estimated from the above data (Figure 2D). These results indicated that MZ B cells were relatively elevated in 3B4/Cre/RBP-J^f/+^ mice and were reduced in Cre/RBP-J^f/f^ mice. The proportion of MZ B cells remained at a normal level in 3B4/Cre/RBP-J^f/f^ mice. Taken together, these data suggested that Notch signaling is required for MZ B cell development in mice with or without transgenic IgH; however, a substantial number of MZ B cells can develop independent of Notch signaling in IgH transgenic mice.

### MZ B cells in 3B4/Cre/RBP-J^f/f^ mice had identical antigen specificity

**Figure 3:**
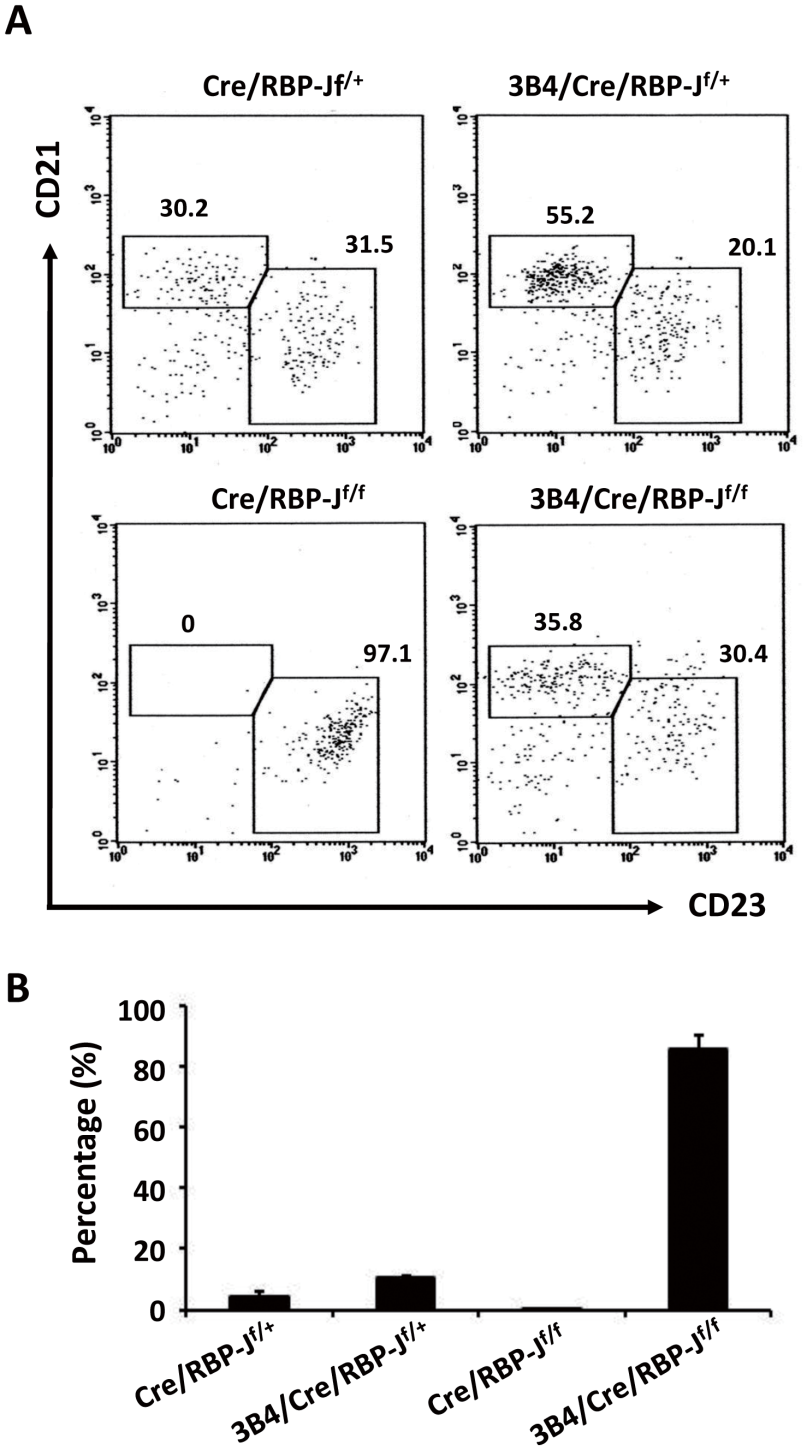
BCR specificity analysis of B cells in the spleen. (A) FITC-conjugated actin in combination with anti-CD21 and anti-CD23 antibodies was used to analyze the antigen specificity of MZ and FO B cells by FCM following BM transplantation. The cells were first gated by actin staining and then analyzed according to CD21 and CD23 expression. (B) The percentage of actin-reactive MZ B cells within the total MZ B cell population is shown. Data are representative of five independent experiments.

To explore the features of the MZ B cells in the absence of Notch signaling, we analyzed the antigen specificity of the MZ B cells in 3B4/Cre/RBP-J^f/f^ mice. As we did not have access to an idiotype-specific antibody against the monoclonal 3B4 antibody, we utilized FITC-conjugated actin to analyze BCR specificity [36], as the antibodies from the 3B4 hybridoma bind actin. The flow cytometry data shown in Figure 3A is the proportion of MZ B and FO B cells gated from actin-positive cells of spleen. Absolute number of actin-bound MZ B cells was then calculated and divided by total MZ B cells to get the data shown in Figure 3B, the percentage of MZ B cells that bound actin. The results showed that the majority of the MZ B cells in 3B4/Cre/RBP-J^f/f^ mice bound actin, while in 3B4/Cre/RBP-J^f/+^ mice only approximately 10% of MZ B cells bound actin (Figure 3A and B). Similar results were obtained when keratin-FITC and myosin-FITC were used to analyze the antigen specificity of the MZ B cells, as 3B4 antibody can also bind these two antigens (data not shown). These results indicated that only MZ B cells with certain antigen specificity could survive the Notch deficiency, and Notch signaling may affect MZ B cell development by interfering with BCR signaling.

### Integration of BCR and Notch signaling occurs at the T2-MZP stage

**Figure 4:**
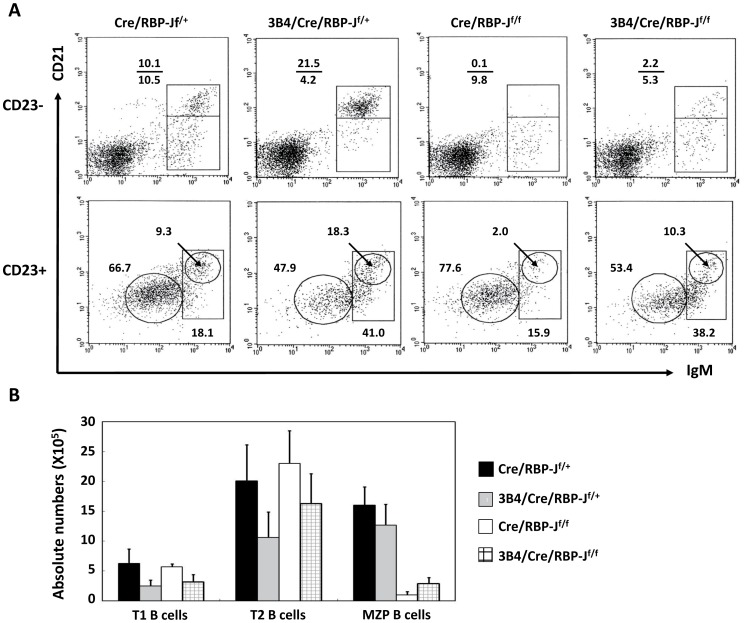
Analysis of transitional B cells in the spleen. (A) Splenocytes were stained with antibodies specific for CD21, CD23 and IgM, and subjected to FCM analysis. CD23^−^ (A) and CD23^+^ (B) populations were gated separately and analyzed according to CD21 and IgM expression. Cell populations were defined using the following markers: T1 cells, CD23^−^IgM^hi^CD21^−^; T2 B cells, CD23^+^IgM^hi^CD21^+^; MZP cells, CD23^+^IgM^hi^CD21^hi^; MZ B cells, CD23^−^IgM^hi^CD21^hi^; and FO B cells, CD23^+^IgM^lo^CD21^lo^. The numbers denote the percentages of cells in the indicated squares. Data are representative of five independent experiments. (B) The absolute numbers of splenic T1, T2 and MZP B cells were calculated from the FCM data in (A) and the total number of erythrocyte-depleted splenocytes. Data represent the mean±S.D. from five independent experiments.

We next sought to determine the developmental stage during which BCR and Notch signaling interact to influence MZ B cell development. To discriminate between T1, T2, MZP, FO and MZ B cells, we analyzed splenic B cell surface expression of IgM, CD21 and CD23. We found that the number of T1 (CD23^−^IgM^hi^CD21^lo^) cells was decreased by approximately one-fold in 3B4/Cre/RBP-J^f/+^ mice and 3B4/Cre/RBP-J^f/f^ mice compared with the Cre/RBP-J^f/+^ mice and Cre/RBP-J^f/f^ mice (Figure 4A and B). These results are consistent with the data in Figure 2B, in which CD21 and CD24 surface expression was utilized for analysis. There was also a slight reduction in the number of T2 (CD23^+^IgM^hi^CD21^int^) cells in 3B4/Cre/RBP-J^f/+^ mice and 3B4/Cre/RBP-J^f/f^ mice compared with the other two groups (Figure 4A and B). Interestingly, the greatest reduction occurred at the MZP (CD23^+^IgM^hi^CD21^hi^) stage. In the Cre/RBP-J^f/f^ mice and 3B4/Cre/RBP-J^f/f^ mice, the number of MZP B cells was less than one fifth of that in the Cre/RBP-J^f/+^ and 3B4/Cre/RBP-J^f/+^ mice (Figure 4A and B). 3B4/Cre/RBP-J^f/f^ mice possessed more MZP cells than Cre/RBP-J^f/f^ mice. However, there was no difference in the number of MZP B cells between Cre/RBP-J^f/+^ and 3B4/Cre/RBP-J^f/+^ mice. These data indicated that in 3B4/Cre/RBP-J^f/+^ mice, the biased development to MZ B cells began at the T2-MZP stage, after this point the development of FO B cells was interrupted while MZP and MZ B cell differentiation remained intact. In Cre/RBP-J^f/f^ mice, the effects of Notch-RBP-J deficiency may also begin at the T2-MZP stage, resulting in the marked reduction of MZP and MZ B cell numbers. In 3B4/Cre/RBP-J^f/f^ mice, while the number of both T2 cells and MZP cells was decreased, the number of MZP and MZ B cells was doubled compared with Cre/RBP-J^f/f^ mice, indicating that abrogation of Notch signaling may interfere with BCR-directed MZ B cell development at the T2-MZP stage.

### Analysis of BM B cell development

**Figure 5:**
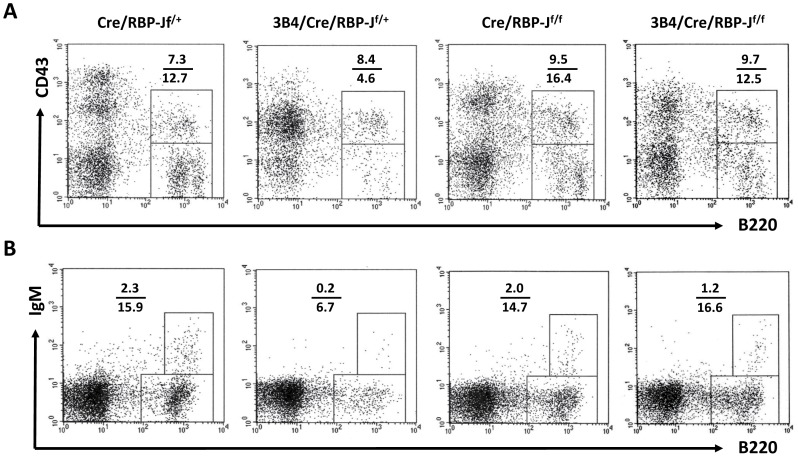
Analysis of BM B cells. BM cells derived from different groups of mice were stained with anti-B220, anti-CD43 and anti-IgM (A), or anti-IgM, anti- B220 and anti-CD23 (B) and analyzed using FCM. (A) Cells were first gated using anti-IgM staining and then IgM^−^ cells were analyzed according to B220 and CD43 expression. Pro-B cells and pre-B cells were defined as being IgM^−^CD43^+^B220^+^ and IgM^−^CD43^−^B220^+^, respectively. (B) Cells were first gated using anti-CD23 and then CD23^−^ cells were analyzed according B220 and IgM expression. Immature B cells were defined as being CD23^−^IgM^+^B220^+^. Data are representative of five independent experiments.

We also analyzed B cell development in the BM following the deletion of the RBP-J allele. When analyzed using anti-CD43, anti-IgM and anti-B220 antibodies to discriminate pro-B (IgM^−^B220^+^CD43^+^) and pre-B (IgM^−^B220^+^CD43^−^) cells, we found a slightly elevated proportion of pro-B cells in Cre/RBP-J^f/f^ mice and 3B4/Cre/RBP-J^f/f^ mice compared with Cre/RBP-J^f/+^ mice and 3B4/Cre/RBP-J^f/+^ mice, indicating that the absence of Notch signaling promote pro-B cells production (Figure 5A). Consistent with our previous study, pre-B cell population in 3B4/Cre/RBP-J^f/+^ mice were decreased to less than one half of the other three strains of mice [36]; this decrease was predominately due to the aberrant paring of the μ chain with the surrogate light chain to form pre-BCR (Figure 5A). However, there was no decrease in the proportion of pre-B cells in the 3B4/Cre/RBP-J^f/f^ mice, indicating that the abrogation of Notch signaling may help B cells to pass through the pre-BCR checkpoint. To analyze newly formed immature B cells (CD23^−^IgM^+^B220^+^), CD23^−^ cells were gated and subsequently identified using anti-IgM and anti-B220 antibodies. We observed a marked reduction in the number of immature B cells in 3B4/Cre/RBP-J^f/+^ mice; further, no differences between Cre/RBP-J^f/+^, Cre/RBP-J^f/f^ and 3B4/Cre/RBP-J^f/f^ mice were noted (Figure 5B). Our data suggests that when pre-BCR signaling is aberrant, loss of Notch signaling can overcome the developmental block in the BM of 3B4/Cre/RBP-J^f/f^ mice.

## Discussion

The aim of the present study was to explore the integration of BCR and Notch signaling in MZ B cell development. B cell development in mice with a transgenic IgH that lack Notch signaling was analyzed by using IgH transgenic mice TgV_H_3B4, RBP-J-floxed mice and Mx-Cre mice. Our data indicated that the elevated population of MZ B cells observed in TgV_H_3B4 mice was greatly reduced following abrogation of Notch signaling in 3B4/Cre/RBP-J^f/f^ mice. These finding were consistent with those obtained from Cre/RBP-J^f/f^ mice, and indicated that Notch signaling is required for BCR-directed MZ B cell development. However, in contrast to Cre/RBP-J^f/f^ mice, a small number of MZ B cells that escaped the Notch-RBP-J checkpoint were observed in 3B4/Cre/RBP-J^f/f^ mice. Further analysis of the BCR specificity of these remaining MZ B cells indicated that these cells exhibited identical antigen specificity derived from the original 3B4 antibody. These data suggested that certain B cells with particular BCR specificity can develop into MZ B cells without Notch signaling. Our study revealed for the first time that MZ B cells can develop in a Notch-independent manner.

Both the BCR and Notch signaling pathways play important roles in B cell development, activation, and lymphomagenesis. However, if and how the BCR and Notch signaling pathways interact with each other remains unclear. Several studies reported that BCR and Notch signaling cooperated to promote B cell activation. He et al. used a soluble human Dll1 fragment and anti-IgM to stimulate Burkitt's lymphoma Raji cells and found that Notch signaling may interact with BCR signaling at the level of c-myc expression to regulate B-lymphoma cell proliferation and apoptosis [38]. Thomas et al. demonstrated that costimulation of follicular B cells with Dll1 leads to significant increases in BCR- and CD40-mediated proliferation and enhances production of IgG1-positive cells *in vitro* and *in vivo*; taken together, these data suggest that the BCR and CD40 signaling pathways collaborate with the Notch pathway to optimize B cell activation [39]. However, the interaction between BCR and Notch signaling in MZ B cell development remains unclear.

Pillai et al. proposed that strong BCR signaling activates Bruton's tyrosine kinase, which inhibits Notch2 signaling and activates the canonical NF-κB signaling pathway, subsequently promoting FO B cell development [29]. During MZ B cell development, weak BCR signaling permits Notch2 signaling. The interaction of Dll1 with Notch2 leads to the activation of MAML1 and RBP-J and MZ B cell development [29]. Pillai hypothesized that Notch2 signaling is the primary driving force for MZ B cell development, and that BCR signaling affects MZ B cell development by interfering with the Notch pathway. However, our study suggested that BCR signaling may be the primary driving force for MZ B cell development, and that Notch signaling may affect the BCR pathway to alter the direction of B cell development. The helix-loop-helix transcription factor E2A is an important candidate molecule that might link the BCR and Notch signaling pathways. E2A^+/−^ mice exhibited increased MZ B cell development at the expense of FO B cell development [40]. Interestingly, Notch signaling can cause the degeneration of both E2A isoforms (E12 and E47) and may therefore contribute to MZ B cell development by regulating the availability of the E12 and E47 transcriptional regulators [41]. More studies are needed to elucidate the detailed mechanisms underlying BCR and Notch interactions.

Although Notch has long been known to regulate MZ B cell development, the stage at which Notch signaling exerts its effects is unknown. The ligand for Notch2, Dll1, is expressed on the luminal face of venules that are predominately found in the red pulp of the spleen, and some Dl1 expression is also present in the MZ [42]. Thus, Notch signaling would be activated when B cells migrate from blood vessels to the MZ or when B cells shuttle between the follicles and the MZ. Our data indicate that the reduction of MZ B cells begins at the T2-MZP stage, indicating that the function of Notch and the interaction between BCR signaling and Notch signaling likely occurred during the T2-MZP stage. This hypothesis is consistent with the finding that T2 B cells are the common precursor of both FO and MZ B cells prior to differentiation [2].

Analysis of BM B cell development revealed a slightly elevated numbers of pro-B cells in Cre/RBP-J^f/f^ mice and 3B4/Cre/RBP-J^f/f^ mice, which is different with that of CD19-Cre/RBP-J^f/f^ mice [18]. The reason may be that Mx-Cre induces complete deletion of RBP-J allele before the pro-B cell satge, while CD19-Cre only partially induces the RBP-J deletion at the pro- and pre-B cell stage. Consistent with previous reports, we also observed reduced numbers of pre-B cells in 3B4/Cre/RBP-J^f/+^ mice, a finding that may be due to the aberrant paring of the μ chain with the surrogate light chain to form the pre-BCR. In the BM, appropriate pairing of the μ chains with surrogate light chains to form the pre-BCR is critical for B cell development prior to surface IgM expression at the pre-B cell stage [43]–[45]. However, the number of pre-B cells in 3B4/Cre/RBP-J^f/f^ mice is normal compared with Cre/RBP-J^f/+^ mice, indicating that the absence of Notch signaling might promote pre-B cell development at the pre-BCR checkpoint.

## Materials and Methods

### Ethics Statements

The animal husbandry, experiments and welfare were conducted in accordance with the Detailed Rules for the Administration of Animal Experiments for Medical Research Purposes issued by the Ministry of Health of China, and were approved by the Animal Experiment Administration Committee of Fourth Military Medical University. Mice were raised in the specific pathogen free conditions on the C57BL/6 background, and were manipulated with every specific care to reduce the suffering of the mice during the experiments.

### Mice

IgH transgenic mice TgV_H_3B4 were generated in our lab; line #39 was utilized in this study because of its high allelic exclusion [35], [36]. RBP-J-floxed mice and Mx-Cre mice have been described previously [37]. TgV_H_3B4 mice were crossed with Mx-Cre/RBP-J^f/f^ mice for at least two generations to obtain mice with the genotypes of Cre/RBP-J^f/+^, 3B4/Cre/RBP-J^f/+^, Cre/RBP-J^f/f^ and 3B4/Cre/RBP-J^f/f^. The transgenic mice were genotyped using PCR as described previously.

### Deletion of the RBP-J gene through Cre-mediated recombination

Mice with genotypes of Cre/RBP-J^f/+^, 3B4/Cre/RBP-J^f/+^, Cre/RBP-J^f/f^, 3B4/Cre/RBP-J^f/f^ and wild-type were employed in the current study. To induce the Cre-mediated deletion of the RBP-J gene, 6- to 8-week-old mice were injected *i.p.* with 300 µg of poly(I:C) (Sigma, St. Louis) for four times at two-day intervals. Mice were then injected with the same amount of poly(I:C) for four additional times at one-week intervals; in total, mice were given eight injections. Two days following the last injection, the mice were sacrificed for further analysis. Southern blot analysis was utilized to determine the deletion efficiency of the RBP-Jκ allele as described previously [37].

### BM transplantation

BM cells were prepared from mice injected with poly(I:C). Recipients were irradiated with 8 Gy and then *i.v.* injected with 2×10^6^ donor BM cells through the tail vein. Mice were maintained on aqueous antibiotics (1.1 g/L of neomycin sulfate) and analyzed after 2 months.

### Flow cytometry

Cells were collected from mouse tissues and treated with buffered 0.14 M NH_4_Cl. Cells (5×10^5^) were stained with antibodies for 30 min on ice and analyzed using a FACSCalibur™ (BD Immunocytometry Systems, San Jose, CA). Data were analyzed using Cellquest™ software. Dead cells were excluded using propidium iodide staining. The antibodies used in the analyses were as follows: anti-B220 (RA3-6B2), anti-IgM (R6-60.2), anti-CD5 (53–7.3), anti-CD19 (1D3), anti-CD21/35 (7G6), anti-CD23 (B3B4), anti-CD24 (M1/69), anti-CD43 (S7), and streptavidin-APC. All antibodies were obtained from BD PharMingen (San Diego, CA). FITC-conjugated actin was prepared according to standardized methods.

### Immunofluorescent staining of tissue sections

Tissue samples from spleens were frozen in tissue-tek OCT compound and cut at a thickness of 6 µm. After air-drying, sections were fixed in 4% paraformaldehyde for 20 min. Immunohistochemical staining was done with Cy3-anti–mouse IgM and FITC-anti–MOMA-1 (Serotec) antibodies for 1 h at room temperature. Slides were analyzed with a laser scanning confocal microscope (OLYMPUS FluoView™ FV1000).

### Data analysis

ANOVA was used to determine the statistical significance of values among experimental groups. The statistical significance was defined as p<0.05.
